# Editorial: Mechanisms underlying plant-pollinator-herbivore interactions

**DOI:** 10.3389/fpls.2022.1033287

**Published:** 2022-09-21

**Authors:** Anne Muola, Dani Lucas-Barbosa, André Kessler

**Affiliations:** ^1^ Biotechnology and Plant Health Division, Norwegian Institute of Bioeconomy Research (NIBIO), Tromsø, Norway; ^2^ Department of Crop Sciences , Research Institute of Organic Agriculture FiBL, Frick, Switzerland; ^3^ Department of Ecology and Evolutionary Biology, Cornell University, Ithaca, NY, United States

**Keywords:** plant defence, pollinator attraction, floral rewards, secondary metabolites, mutualism, antagonism, plant fitness

The success of a particular plant phenotype in any given environment depends on how well the expression of traits allows plants to negotiate interactions with other organisms. Because plants cannot move away from unfavorable conditions, they express traits that attract mutualists and repel antagonists. These interaction-mediating traits have been well studied for plant interactions with herbivores as antagonists and pollinators as mutualists, to an extent that they both became models for understanding biotic interactions in general. Recently, these two schools of thought, plant defense theory and pollinator attraction have started to merge as scientists became aware that many of the plant traits that mediate the respective interaction, are overlapping. So, both fields of research face the same fundamental question: How do plants attract mutualists and repel antagonists with the same suit of basic traits, much relying on secondary metabolites, and within the same information space?

This conflict that plants face is most apparent when considering chemical traits mediating biotic interactions. Plants can produce toxic, anti-digestive and anti-nutritive compounds that directly protect them against herbivores and pathogens and/or that are induced following the attack. Moreover, the herbivory-induced production of volatile organic compounds can attract predators and parasitoids facilitating the top-down control of pest organisms (indirect defenses). Thus, plant secondary metabolism allows plants to limit the number of potential antagonistic interactors (constitutive defenses) and to involve the entire interaction community in a plant defense strategy (information transfer through induced responses; Kessler, 2015). This, however, includes the interaction with mutualist pollinators as well.

Although plant chemistry had been suggested to be important in pollinator attraction for a long time, actual experimental proof was provided relatively recently (Raguso, 2008). Pollinator rewards (e.g. nectar, pollen, oils) include many of the same defensive compounds as leaves do, consequentially exposing pollinators to those same traits and suggesting pollinators as potential agents of natural selection on plant defense traits (Adler, 2000). The multifunctionality of secondary metabolites suggests diffuse reciprocal natural selection on plant secondary metabolism by the pollinator and herbivore communities associated with a plant as well as the interactor’s ability to cope with plant chemical defenses (Ramos and Schiestl, 2019). In addition, this means that plant defense strategy and mating systems can be expected to impact ecological interactions and evolutionary paths because of metabolic changes in response to interactions with herbivores or pollinators. Indeed, phylogenetic correlations between defense strategies and mating systems have recently been revealed in comparative studies with Solanaceae (Adler et al., 2012; Campbell and Kessler, 2013). Moreover, plant phenotypic changes induced by herbivory have been found to affect pollinator behavior, often with significant effects on plant fitness (Kessler et al., 2011; Muola et al., 2017). Despite these findings, the mechanisms as well as the evolutionary consequences underlying the observed ecological consequences of a shared information space between plants, pollinators, and herbivores, remain poorly understood (Lucas-Barbosa, 2016; Rusman et al., 2019).

With this special issue we aim to feature impactful developments in the field to identify the paths forward.

Challenges of attracting mutualists while repelling antagonists are likely to be particularly strong with pollinating herbivores, such as many Lepidopteran species with herbivorous larvae and pollinating adults. Jacobsen and Raguso (2021) show that defensive leaf volatiles play a role in host plant selection of the pollinating herbivore *Manduca sexta* and demonstrate that adults assess information from leaves differently when choosing between foraging and oviposition locations. This raises the interesting question if such ontogenetically differential use of chemical information is common or a specific feature of herbivores that also function as major pollinators on their host plants and what drives the evolution of such differential behavior.

Davidowitz et al. (2022) offer a special perspective and partial answer to the latter question. They also focused on pollinating herbivores to explore the hypothesis that increased allocation of resources to flight could lead to higher pollination efficiency of Lepidopteran pollinators, and thus, higher plant fitness. Reciprocally, this also means that if the same insect increases resource allocation to reproduction instead of flight, herbivore population size is likely to increase with potentially negative consequences for plant fitness. Davidowitz et al. propose such resource allocation trade-offs between flight and fecundity in insects as potential drivers of differential selection on plant defenses and pollinator, herbivore counter defenses, as well as plant reproductive traits. For example, Villamill et al. (2022) show how herbivory can affect plant sexual expression by teasing apart the effects of herbivory-caused resource limitation and plant responses to herbivory-induced jasmonate signaling on wind-pollinated *Mercurialis annua*. They found that the jasmonate-mediated induction reverses the effect of tissue loss on male reproductive investment indicating that plant defence expression can have consequences for sex allocation and eventually for the evolution of plant sexual systems.

Plant-visiting ants are often functional herbivores or predators, only rarely pollinators. However, Aranda-Rickert et al. (2021) provide the first evidence of distance-dependent contribution of ants to pollination, but with no effect on plant defence. They show how ants can offset pollen limitation in isolated females of wind-pollinated *Ephedra triandra* by contributing to targeted delivery of airborne pollen while they consume the sugary pollination drops. Whether other dioecious plants lacking male reward for pollinators share similar mechanisms of ambophily remains to be studied. Pollinators and herbivores can exert selection on plant reproductive and defensive traits suggesting conflicting selection pressures. Here, Wu et al. (2021) demonstrate that herbivore-mediated selection can generate selective pressures for greater flower production on insect-pollinated plants. Their study indicates that the variation in the intensity of plant-antagonistic interactions can drive spatial variation in natural selection on floral traits.

Despite recent advances, the evolutionary consequences of the complex ecological interactions remain poorly understood. The Review by De-la-Cruz et al. (2022) explains how the traditional experimental and modern methods including next-generation sequencing, metabolomics, and gene-editing technologies can enhance our understanding of the genes and traits involved in mediating complex plant-pollinator-herbivore interactions.

We hope that you enjoy this Research Topic and that it gives inspiration for more research uniting the traditionally separately studied fields of plant-pollinator and plant-herbivore interactions.

## Author contributions

All authors listed have made substantial intellectual contributions to the work and approved it for publication.

## Conflict of interest

The authors declare that the research was conducted in the absence of any commercial or financial relationships that could be construed as a potential conflict of interest.

## Publisher’s note

All claims expressed in this article are solely those of the authors and do not necessarily represent those of their affiliated organizations, or those of the publisher, the editors and the reviewers. Any product that may be evaluated in this article, or claim that may be made by its manufacturer, is not guaranteed or endorsed by the publisher.

## References

[B1] Adler L. S. (2000). The ecological significance of toxic nectar. Oikos 90, 409–420. doi: 10.1034/j.1600-0706.2000.910301.x

[B2] Adler L. S. Seifert M. G. Wink M. Morse G. E. (2012). Reliance on pollinators predicts defensive chemistry across tobacco species. Ecol. Lett. 15, 1140–1148. doi: 10.1111/j.1461-0248.2012.01838.x 22834564

[B3] Campbell S. A. Kessler A. (2013). Plant mating system transitions drive the macroevolution of defense strategies. Proc. Natl. Acad. Sci. U. S. A 110, 3973–3978. doi: 10.1073/pnas.1213867110 23431190 PMC3593862

[B4] Kessler A. (2015). The information landscape of plant constitutive and induced secondary metabolite production. Curr. Opin. Insect Sci. 8, 47–53. doi: 10.1016/j.cois.2015.02.002 32846677

[B5] Kessler A. Halitschke R. Poveda K. (2011). Herbivory-mediated pollinator limitation: Negative impacts of induced volatiles on plant–pollinator interactions. Ecology 92, 1769–1780. doi: 10.1890/10-1945.1 21939073

[B6] Lucas-Barbosa D. (2016). Integrating studies on plant–pollinator and plant–herbivore interactions. Trends Plant Sci. 21, 125–133. doi: 10.1016/j.tplants.2015.10.013 26598297

[B7] Raguso R. A. (2008). Wake up and smell the roses: The ecology and evolution of floral scent. Annu. Rev. Ecology Evolution Systematics 39, 549–569. doi: 10.1146/annurev.ecolsys.38.091206.095601

[B8] Ramos S. E. Schiestl F. P. (2019). Rapid plant evolution driven by the interaction of pollination and herbivory. Science 364, 193–196. doi: 10.1126/science.aav6962 30975889

[B9] Rusman Q. Lucas-Barbosa D. Poelman E. H. Dicke M. (2019). Ecology of plastic flowers. Trends Plant Sci. 24, 725–740. doi: 10.1016/j.tplants.2019.04.007 31204246

